# Efficacy and Safety in the Continued Treatment With a Biosimilar Drug in Patients Receiving Infliximab: A Systematic Review in the Context of Decision-Making From a Latin-American Country

**DOI:** 10.3389/fphar.2019.01010

**Published:** 2019-11-15

**Authors:** Edward Mezones-Holguin, Rocio Violeta Gamboa-Cardenas, Gadwyn Sanchez-Felix, José Chávez-Corrales, Luis Miguel Helguero-Santin, Luis Max Laban Seminario, Paula Alejandra Burela-Prado, Maribel Marilu Castro-Reyes, Fabian Fiestas

**Affiliations:** ^1^Universidad San Ignacio de Loyola (USIL), Centro de Excelencia en Estudios Económicos y Sociales en Salud, Lima, Peru; ^2^Seguro Social en Salud (EsSalud), Hospital Nacional Guillermo Almenara Irigoyen, Servicio de Reumatologia, Lima, Peru; ^3^Seguro Social en Salud (EsSalud), Hospital Nacional Edgardo Rebagliati Martins, Servicio de Dermatología, Lima, Peru; ^4^Seguro Social en Salud (EsSalud), Hospital Nacional Edgardo Rebagliati Martins, Servicio de Reumatologia, Lima, Peru; ^5^Universidad Nacional de Piura (UNP), Facultad de Ciencias de la Salud, Sociedad Científica de Estudiantes de Medicina (SOCIEMUNP), Piura, Peru; ^6^Seguro Social en Salud (EsSalud), Instituto de Evaluación de Tecnologías Sanitarias e Investigación (IETSI), Lima, Peru

**Keywords:** Infliximab, biosimilar, interchangeability, decision making, Latin-American

## Abstract

**Introduction:** Biological products, including infliximab (INF), are a therapeutic option for various medical conditions. In the Peruvian Social Security (EsSalud), infliximab is approved for the treatment of rheumatoid arthritis, psoriasis, psoriatic arthropathy, ankylosing spondylitis, ulcerative colitis and Crohn’s disease (in cases refractory to conventional treatment). Biosimilars are a safe and effective alternative approved for these diseases in patients who start treatment with infliximab. Nevertheless, there are people in treatment with the biological reference product (BRP), in whom the continuing therapy with a biosimilar biological product (BBP) must be evaluated.

**Objectives:** To synthesize the best available evidence, calculate a preliminary financial impact and conduct technical discussions about the interchangeability into biosimilar in patients receiving treatment with original infliximab for medical conditions approved in EsSalud.

**Methodology:** We carried out a systematic review of controlled clinical trials. Primary search was performed in Pubmed- MEDLINE, SCOPUS, WOS, EMBASE, TRIPDATABASE, DARE, Cochrane Library, NICE, AHRQ, SMC, McMaster-PLUS, CADTH, and HSE until June-2018. We used the Cochrane Collaboration tool to assess the risk of bias. Also, we implemented a preliminary financial analysis about the impact of biosimilar introduction on institutional purchasing budget. Moreover, technical meetings with medical doctors specialized in rheumatology, gastroenterology and dermatology were held for discussing findings.

**Results:** In primary search, 1136 records were identified, and 357 duplicates were removed. From 799 records, we excluded 765 after title and abstract evaluation. From 14 full-text appraised documents, we included five clinical trials in the risk of bias assessment: four studies evaluated CTP-13 and one tested SB2. Two double-blind clinical trials reported no differences in efficacy and safety profiles between maintenance group (INF/INF) and interchangeability group in all diseases included (INF/CTP-13) and rheumatoid arthritis (CTP13 and SB2). In the other three studies, open-label extension of primary clinical trials, no differences were founded in efficacy and safety profiles between CTP-13/CTP-13 and INF/CTP-13 groups. In financial analysis, the inclusion of biosimilars implied savings around S/7´642,780.00 (1USD=S/3.30) on purchasing budget of EsSalud. In technical meetings, beyond certain concerns, specialists agreed with the findings.

**Conclusions:** Evidence from clinical trials support that there are no differences in efficacy or safety of continuing the treatment with Infliximab BRP or exchanging into its biosimilar in patients with medical conditions approved in EsSalud. Financial analysis shows that the biosimilar introduction produce savings in purchasing institutional budget. Therefore, based on cost-opportunity principle, exchanging into biosimilar in patients receiving the original Infliximab, is a valid therapeutic alternative in the Peruvian Social Security.

## Introduction

Biological products are therapeutic options for different diseases. These drugs are molecules with a complex structure, large and often highly specific and are derived from living organisms (Pombo et al., 2009; Wang and Singh, 2013; Auclair, 2019). Biologic drugs are used to treat various diseases, including conditions that involve the immune system, randomized studies have shown their efficacy for reducing symptoms and improving the quality of life in people undergoing treatment (Wang and Singh, 2013; Zelikin et al., 2016). However, a significant number of patients do not respond, have an inadequate response to initial treatment (primary failure), lose response over time (secondary failure), or may develop adverse effects potentially limiting the therapy (Auclair, 2019). One of these drugs is infliximab (REMICADE^®^), a tumor necrosis factor alpha inhibitor (TNFa) (Ecker et al., 2015). Infliximab has been approved for the treatment of rheumatoid arthritis (RA), severe psoriasis, ankylosing spondylitis, Crohn’s disease, and ulcerative colitis, among other diseases.(Acevedo and Gaitan, 2012; European Medicines Agency (EMA), 2018; Food and Drug Administration, 2018). Efficacy, effectiveness, and safety of this biological drug has been tested in different studies (Li et al., 2017). Hence, infliximab is currently included in the pharmacological petition of the Peruvian Social Security (EsSalud). (Seguro Social en Salud (EsSalud), 2017).

On the other hand, the biosimilar biological products (BBP) are an efficient treatment alternative to the biological reference products (BRP). They usually offering similar effects and lower cost (Declerck et al., 2017; Gutka et al., 2018). BBP contains the active component of BRP with similar characteristics in its pharmacological activity, efficacy and safety (Gamez-Belmonte et al., 2018; Gutka et al., 2018). The equivalence of BBP has been reported from comparison – in equal terms - with BRP in various randomized clinical trials (Portela et al., 2017; Uhlig and Goll, 2017), where infliximab is one the most studied drugs (Gutka et al., 2018). Based on this information, international guidelines for its regulation have been spread and adopted in several countries (Garcia and Araujo, 2016; Sheets, 2017; Tsai, 2017; Zahl, 2017). The Peruvian health system is fragmented, segmented and inequitable (Sánchez-Moreno, 2014), where around 25% of population are affiliated to EsSalud (Mezones-Holguin et al., 2019). The General Directorate of Medicines, Supplies and Drugs (DIGEMID, from Spanish Acronym) as the national health authority, approved the commercialization of some infliximab biosimilars in Peru (Ministerio de Salud, Dirección General de Medicamentos, Insumos y Drogas (DIGEMID), 2016). Therefore, certain public institutions, supported by the Peruvian Government contracting laws, including the Social Security, have purchased BBP. Currently, in EsSalud there are two kinds of patients: those who will start treatment with Infliximab and those who continue their therapy with Infliximab. In the first group, the use of biosimilar is accepted as valid; however, in the other group, there are certain concerns with respect to the continuation with BBP.

Based on the context described, a decision should be made regarding the continuation with a biosimilar in patients undergoing treatment with original infliximab in EsSalud. Although, there are several definitions on interchangeability, in our manuscript it means a transition from using BRP to BBP (Gutka et al., 2018; Trifirò et al., 2018). At the moment, there is an interesting debate about interchangeability with active participation of distinct actors from different health care systems; thus, international regulations have been proposed to the use of BBP and the transition from its BRP (Portela et al., 2017; Tsai, 2017; Cohen et al., 2018; Niazi, 2018). Nevertheless, in Peru, and specifically in EsSalud, there is no explicit decree for it. Therefore, the Institute for Health Technology Assessment and Research (IETSI, from Spanish acronym) - as technical entity in EsSalud - must evaluate the best available scientific evidence to inform decision-making in the Peruvian Social Security.

In light of the above mentioned, the aim of our study was to synthesize the best available evidence, calculate a preliminary financial impact, and conduct a technical discussion concerning the interchangeability into biosimilar in patients undergoing treatment with original infliximab for medical conditions approved in EsSalud. Although there are systematic reviews published (Chingcuanco et al., 2016; Cohen et al., 2018; McKinnon et al., 2018; Feagan et al., 2019), our study incorporates two key elements used in the decision-making process for health systems with limited resources: institutional budget and clinical experience. Consequently, our article is a description of this complexity in Peru and shows the use of the best scientific evidence in the real world.

## Methods

In our manuscript, we describe the three main activities performed in order to inform the decision-making process in EsSalud regarding infliximab interchangeability:

a) Systematic review based on PRISMA guidelines (Moher et al., 2009),
b) Preliminary financial analysis about the direct impact on institutional purchasing budget of EsSalud, and
c) Technical meeting with rheumatologists, dermatologists and gastroenterologists for discussing the results from clinical practice perspective.

## Systematic Review

### Clinical Question (PICOS)

The population(P) was circumscribed to adults with rheumatoid arthritis, psoriasis, ulcerative colitis, Crohn’s disease and ankylosing spondylitis undergoing treatment with the original Infliximab. Intervention(I) was to exchange into a biosimilar, and comparison(C) was the continuation with original Infliximab. The outcomes(O) were efficacy and safety. In accordance with current legal regulations in EsSalud, we included only controlled clinical studies(S) in biosimilar drugs approved by DIGEMID for their commercialization in Peru (CTP-13 and SB2).

### Search Strategy and Selection of Study

We conducted a search without language restrictions until June 2018. Primary strategy formulation included controlled and free terms according to PICOS question. Studies were restricted to clinical trials in humans of any age, gender or nationality. We searched in: PubMed-MEDLINE, SCOPUS, Web of Science (WOS), Excerpta Medica (EMBASE), Translating Research into Practice (TRIPDATABASE), Database of Abstracts of Reviews of Effects (DARE), Cochrane Central Register of Controlled Trials (CENTRAL), National Institute for Health and Care Excellence (NICE), The Agency for Healthcare Research and Quality (AHRQ), The Scottish Medicines Consortium (SMC), McMaster PLUS, The Canadian Agency for Drugs and Technologies in Health (CADTH), and The Health Systems Evidence (HSE). Primary search strategies for each database are explicitly presented as annexes (A-N) (**Supplementary Table 1**). Additionally, we reviewed the list of references. Poster and oral presentations in scientific meetings were not considered.

### Article Selection

Records found were collected in an electronic folder using Mendeley^®^ (Elsevier Inc, NY, USA) and we generated a Research Information Systems (RIS) file. Duplicates were removed by automatic and manual methods; then, we exported a new file to Rayyan^®^ (Qatar Computer Research Institute, Doha, Qatar). Two authors (LHS and LLS) completed a blind and independent selection based on abstract and title, third author (EMH) had diriment decision. Then, two authors (LH-S and LL-S) selected articles in full-text evaluation with third author as diriment (EM-H). Afterward, two evaluators (LHS and LLS) codified the articles and uploaded them in Google Drive^®^ folder (Google Inc, CA, USA).

### Risk of Bias Assessment

Two authors (LHS and LLS) acted upon blind and independent appraisal of clinical trials using the Cochrane Collaboration tool(Higgins et al., 2011). Disagreements were resolved by consensus and diriment participation (EMH).

### Statistical Synthesis

Although a meta-analysis was initially proposed, it was not performed due to clinical and methodological heterogeneity.

### Preliminary Financial Analysis

We implemented an analysis about the impact of biosimilar introduction in the institutional purchasing budget based on the official reports of EsSalud and Electronic Government Procurement System of Peru (SEACE, from Spanish Acronym).

### Technical Meeting

We held several face-to-face meetings to present and discuss the results of the systematic review and financial analysis. A group of rheumatologists, dermatologists and gastroenterologists working in hospitals of EsSalud in Lima, participated in these reunions.

## Results

### Selection and Characteristics of Studies

**Figure 1:**
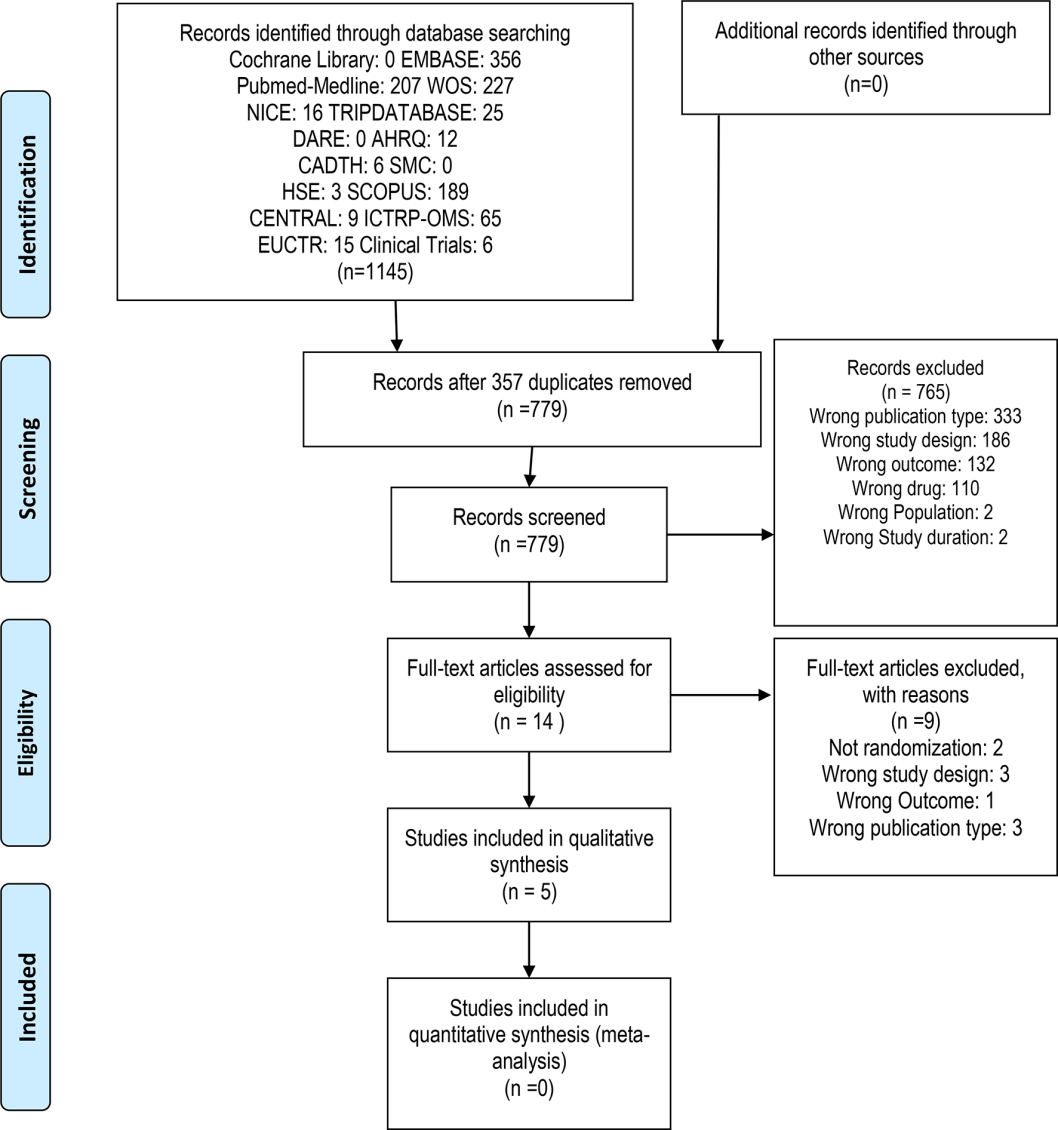
Flow diagram of Study Selection according PRISMA guidelines.

We identified a total of 1136 records in the primary search, from which we removed 357 duplicates. From 799 screened records, we excluded 765 in title and abstract evaluation. Then, we appraised 14 full-text documents, and included five clinical trials for risk of bias assessment and data extraction (**Figure 1**).

We found five controlled studies, that corresponded to five publications, which evaluated the interchangeability between Infliximab and its PBB. Only one assessed SB2 biosimilar (Smolen et al., 2018), and the four remaining studies evaluated biosimilar CTP-13. Two publications were double-blind Randomized controlled studies (RCT), while the remaining three were open-label continuation of clinical trials that initially compared the PBR with PBB. Three studies focused specifically on patients with rheumatoid arthritis, one in ankylosing spondylitis and another, in addition to these two diseases, included Crohn’s disease, ulcerative colitis, psoriatic arthritis and chronic plaque psoriasis. In **Table 1** we present the general characteristics of trials included.

**Table 1:** Characteristics of primary studies included in the analysis.

Author (Year)	Design (Founding)	Population	Countries	Comparison (Pre/post exchange)*	Average time (Pre/post exchange)	Conclusion
***Biosimilar: SB2***						
Smolen et al. (2018)	Randomized double-blind phase 3 trial(Samsung Bioepis Co Ltd.)	Rheumatoid arthritis	Bulgaria Colombia Czech Republic Hungary Republic of Korea Lithuania Mexico Poland Ukraine UK	INF/INF (n=101) SB2/SB2 (n=201) INF/SB2 (n=92)	(54/46 weeks)	The efficacy, safety and immunogenicity profiles were similar between the groups: INF/SB2,INF/INF and SB2/SB2.No emergent treatment or clinically relevant problems were observed after the change from INF to SB2
***Biosimilar: CT-P13***						
Jørgensen et al. (2017)	Randomized double-blind non-inferiority phase 4 trial(Government of Norway)	Crohn’s disease, ulcerative colitis, rheumatoid arthritis, spondylarthritis, psoriatic arthritis, chronic plaque psoriasis	Norway	CT-P13/ CT-P13 (n=241) INF/CT-P13 (n=241)	(26/52 weeks)**	The change from INF to CT-P13 showed no inferiority to the continuous treatment with INF in terms of safety and immunogenicity for all the diseases studied.However, there was not enough statistical power to demonstrate non-inferiority for each disease.
Tanaka (2017)	Open label extension of phase 2 trial(Celltrion Inc)	Rheumatoid arthritis	Japan	CT-P13/ CT-P13 (n=38) INF/CT-P13 (n=33)	(52/72 weeks)***	CT-P13 was well tolerated with persistent efficacy for both groups. Likewise, stable clinical efficacy was shown in patients with RA.
Yoo et al. (2017)	Open label extension of the phase 3-PLANETRA trial(Celltrion Inc)	Rheumatoid arthritis	Bosnia Bulgaria Chile Colombia Italy Latvia Lithuania Mexico Peru Poland Philippines Romania Slovakia Spain UK Ukraine	CT-P13/ CT-P13 (n=158) INF/CT-P13 (n=144)	(54/48 weeks)	The efficacy and tolerability observed was similar between patients who were switched from INF to CTP-13 and those who had a long-term treatment with CT-P13 for two years.
Park et al.(2017)	Open-label extension of a phase 3-PLANETAS trial(Celltrion Inc)	Ankylosing spondylitis	Bulgaria Chile Colombia Republic of Korea Latvia Mexico Poland Portugal Spain Ukraine	CT-P13/ CT-P13 (n=388) INF/CT-P13 (n=86)	(54/48 weeks)	The exchange from the original biological reference product into biosimilar is possible without negative effects on safety and efficacy in patients with ankylosing spondylitis.

* The number of patients corresponds to exchanging started time.

** Randomization was applied in patients who already had treatment with the original infliximab drug for a minimum of 6 months.

*** The initial phase of treatment ended at 54 weeks. The first dose of the second stage started eight weeks later in week 62.

Only two articles respond directly to the PICO question, since they evaluated the exchange of the original Infliximab to the biosimilar compared to the maintenance of the original biotherapy: Smolen et al. (2018) and Jørgensen et al. (2017), who tested SB2 and CTP-13, respectively. In both cases, they did not find statistical differences in efficacy or safety between maintenance and exchanging groups.

The other three publications did not respond directly to PICOS question. These studies did not contain primary safety or efficacy data in a blind setting. Instead, they provided complementary information with the purpose of expanding the perspective of clinical use in potential EsSalud scenarios. Those publications reported the evaluation of open-label continuation of primary clinical trials: Tanaka et al. (2017), Yoo et al. (2017), and Park et al. (2017). In these publications no differences were found in efficacy or safety between patients who switched from original infliximab to biosimilar (INF/CTP-13), and maintained biosimilar treatment (CTP-13/CTP-13).

### Risk of Bias

In **Table 2**, we show the appraisal for each study included. Trials with direct response to PICOS question had lower risk of bias, mainly due to randomization and blinding.

**Table 2:** Risk of bias assessment in each study according Cochrane Collaboration Tool.

Author (Year)	Selection Bias		Performance bias	Detection bias	Attrition bias	Reporting bias	Others
	Randomization	Allocation concealment	Blinding of participants and staff	Blinding of outcome assessors and results	Monitoring, exclusion and abandonment	Selective reporting of results	Other biases
Smolen et al. (2018)*	*Low*	*Low*	*Low*	*Low*	*Low*	*Low*	*Low*
Jorgensen et al. (2017)*	*Low*	*Low*	*Low*	*Low*	*Low*	*Low*	*Low*
Tanaka (2017)	*High*	*High*	*High*	*High*	*Low*	*Low*	*Low*
Yoo et al. (2017)	*High*	*High*	*High*	*High*	*Low*	*Low*	*Low*
Park et al. (2017)	*High*	*High*	*High*	*High*	*Low*	*Low*	*Low*

* These articles respond directly PICOs question.

### Description of Evidence

We briefly described efficacy and safety outcomes for each study: one for SB2 and four for CTP-13. We describe efficacy and safety outcomes.

#### Biosimilar SB2

Smolen et al. (2018) “**Safety, Immunogenicity And Efficacy After Switching From Reference Infliximab To Biosimilar SB2 Compared With Continuing Reference Infliximab And SB2 In Patients With Rheumatoid Arthritis: Results Of A Randomized, Double-Blind, Phase III Transition Study.**” **Annals Of The Rheumatic Diseases; 7:234-40**.

A randomized, double-blind, phase 3 clinical study was carried out in people with rheumatoid arthritis. This study had two initial groups. Patients were randomized into two groups for 52 weeks: 293 were treated with Infliximab (INF) and 292 received biosimilar (SB2). Then, a new randomization was performed, INF group was divided into a maintenance group (INF/INF n=101) or exchanging group (INF/SB2 n=94). Meanwhile, the group initially assigned to SB2 continued with biosimilar (SB2/SB2 n=201). Efficacy, safety and immunogenicity profiles were not different among the groups up to week 78.

### Efficacy

The major findings of this study are presented in **Table 3A**. We describe the findings according the clinical scale used.

**Table 3A:** Efficacy outcomes in patients with rheumatoid arthritis.

Author (Year)	Time	Groups (patients allocated)	ACR20* n(%)	ACR50 n(%)	ACR70 n(%)	DAS28 (media±ds)	EULAR n (%)
Smolen et al. (2018)	Exchange: Week 54 End: Week 78	INF/INF (n=101)	*End:* 68.8%	*End:* 47.3%	*End:* *31.2%*	*Baseline:* 4.1±1.5 End:**	*End* *(93 patients):* *No response: 14 (15.1%)Moderate: 47 (50.5%)* *Good: 32 (34.4%)*
		INF/SB2 (n=94)	*End:* 63.5%	*End:* *37.6%*	*End:* *22.4%*	*Baseline:* 3.9±1.3 End:**:	*End* *(85 patients):* No response: 13 (15.3%)Moderate: 44 (51.8%) Good: 28 (32.9%)
		SB2/SB2 (n=94)	*End:* 68.3%	*End:* 40.6%	*End:* 25.6%	*Baseline:* 4.0±1.4 End:**:	*End* *(180 patients):* No response: 23 (12.8%) Moderate: 93 (51.7%) Good: 64 (35.6%)
	*Estimated p-value*		*p=0.7316*	*p=0.3249*	*p=0.3071*	*NA*	P=0.8074***
Tanaka et al. (2017)	Exchange: Week 62 End: Week 167	CT-P13/CTP-13 n=38	*End:* 29(78.4%)	*End:* *26* (70.3%)	*End:* 20(54.1%)	*Baseline:* -2.66 ± 1.57 *End:* -2.78 ± 1.59	*End:* Moderate or Good: 31(83.8%)
		INF/CTP-13 n=33	*End:* 62.5%)	*End:* 17(53.1%)	*End:* *13* (40.6%)	*Baseline:* -2,01 ± 1.33 *End* -2,03 ± 1.73	*End:* Moderate or Good: 22 (68.8%)
	*Estimated p-value*		*P=0.1535*	*P=0.14*	*P=0.26*	*P=0.612*	P=0.1498***
Yoo et al. (2017)	Exchange: Week 54 End: Week 102	CT-P13/CTP-13 n=168	*End:* 117 (74.1%)	*End:* 78 (49.4%)	*End:* 39(24.7%)	*Baseline:* -2,40±1.27 *End:* -2.40 ± 1.42	*End:* No response: 15 (9.9%)Moderate: 80 (52.6%) Good: 43 (28.3%)
		INF/CTP-13 n=144	*End:* 111(77.1%)	*End:* 78 (54.2%)	*End:* *38 (*26.4%)	*Baseline:* -2.37±1.22 *End:* -2,48±1.43	*End:* No response: 12 (8.5%) Moderate: 69 (48.6%) Good: 46 (32.4%)
	*Estimated p-value*		*p=0.54*	*p=0.40*	*p=0.7341*	*p=0.99*	p=0.669***

ACR20, ACR50 y ACR70: Improvement in 20%, 50% and 70% according to the American College of Rheumatology criteria.

DAS28, Score of activity of the disease in 28 joints with reactive protein C (PCR).

EULAR, European League against Rheumatism.

*Jorgensen et al. study included patients with rheumatoid arthritis, however, the random assignment and the sample calculation were for all pathologies. Because it was a subgroup analysis, no results were reported for RA in this table. Baseline corresponds to time of exchanging.

**No differences were founded between the DAS28 indices for each group, no point values were reported at the end of follow-up. The article did not report any differences using graphic methods.

***Comparison for moderate or good classification.

**Table 3B:** Efficacy findings in clinical trials in patients with ankylosing spondylitis.

Author (Year)	Start / end time	Groups (Patients allocated)	ASAS20 n(%)	ASAS40 n(%)	ASAS PR n(%)	BASDAI (mean)	BASFI (mean)	ASDAS Global Score (mean)	BASMI (mean)
Park et al.(2017)	*Exchange:* Week 54	CT-P13/CT-P13 (n=88)	*End:* 67/83 (80.7)	*End:* 53/83 (63.9)	*End:* 16/83 (19.3)	*End:* 3.19	*End:* 3.24	*End:* 1.86	*End:* 2.4
	*End:* Week 102	INF/CT-P13 (n=86)	*End:* 60/78 (76.9)	*End:* 48/78 (61.5)	*End:* 18/78 (23.1)	*End:* 3.23	*End:* 3.25	*End: 1.97*	*End:* 2.6
		*Estimated p value*	*0.506*	*0.672*	*0.275*	NS*	NS*	NS*	NS*

*ASAS*, The Assessment of Spondylarthritis International Society; *PR*, Partial Remission; *BASDAI*, The Bath Ankylosing Spondylitis Disease Activity Index; *BASFI*, Bath Ankylosing Spondylitis Functional Index; *ASDAS*, Ankylosing Spondylitis Disease Activity Score; *BASMI*, Bath Ankylosing Spondylitis Metrology Index; *ASDAS*, Ankylosing Spondylitis Disease Activity Score.

* Standard deviation was not reported. Authors only compared graphically.

#### American College Of Rheumatology (ACR20, ACR50 And ACR70)

Authors found that the percentage of patients who showed a 20% improvement (ACR20) at week 78 of follow-up was not statistically different between the three groups: INF/INF (68.8%), SB2/SB2 (65.7%) and INF/SB2 (63.5%) (p-value:0.7316). Also, there was no statistically significant difference between groups in the proportion of patients with 50% improvement (ACR50) (p-value:0.3249). Moreover, in 70% improvement (ACR70), no significant differences were found in (p-value: 0.3071): INF/INF group (31.2%), SB2/SB2 (25.6%) and INF/SB2 (22.4%).

#### European League Against Rheumatology Score (EULAR)

EULAR response criteria scores were measured at week 78, no statistically significant differences were observed. Good or moderate responses were 84.9% in INF/INF group, 87.3% in SB2/SB2 group and 84.7% in INF/SB2 group (p-value: 0.8074). Regarding the proportion of patients with good response, there was no significant difference between groups: INF/INF (34.4%), SB2/SB2 (35.6%) and INF/SB2 (32.9%) (p= 0.8740).

#### Diseases Activity Score 28 (DAS28), Simple Disease Activity Index (SDAI) And Clinical Diseases Activity Index (CDAI)

These three instruments were used to measure the activity of the disease and there were no significant statistical differences between randomized groups. DAS28 values were (mean±sd): INF/INF (4,1±1,5), SB2/SB2 (4,0±1,4), y INF/SB2 (3,9±1,3). SDAI score in each group were: INF/INF (15,2±12,0), SB2/SB2 (initial 14,6±12,2), and INF/SB2 (13,2±10,0). Regarding CDAI, patients obtained similar scores: INF/INF (15,2±12,0), SB2/SB2 (initial 14,6±12,2), and INF/SB2 (13,2±10,0). No point values were reported at the end of the follow-up at week 78; authors showed graphically the evolution of the scores during the post interchange period, there is no difference between the three groups evaluated (**Table 3A**).

### Safety

#### Adverse Events

No differences were observed in the frequency of adverse events (AE) among the three post-exchange groups: Specifically, for any AE were: INF/INF (35,6%), SB2/SB2 (40,3%), and INF/SB2 (36,2%) of patients presenting any AE (p=0.546). Regarding serious AE post-exchange, frequencies were: 6.4% in INF/SB2, 3% in INF/INF and 3.5% in SB2/SB2 (p=0.456). Similarly, no differences were found in the frequency of discontinuation due to AE (p=0.625) (**Table 3E**).

#### Immunogenicity

Post-exchange immunogenicity levels were very similar among groups: INF/INF (14.9%), SB2/SB2 (14.1%) and INF/SB2 (14.6%) (p=0.98) (p=0.98) (**Table 3E**).

#### Biosimilar CTP-13

Jørgensen et al., 2017. **“Switching from Originator Infliximab to Biosimilar CT-P13 Compared with Maintained Treatment with Originator Infliximab (NOR-SWITCH): A 52-Week, Randomized, Double-Blind, Non-Inferiority Trial.” Lancet 389 (10086): 2304–16** (Jørgensen et al., 2017).

The authors conducted a phase 4, randomized double-blind non-inferiority trial. Patients with Crohn’s disease, ulcerative colitis, rheumatoid arthritis, spondylarthrosis, psoriatic arthritis and chronic plaque psoriasis receiving original infliximab were enrolled and randomized in two arms:: maintenance group with the original biological component (INF/INF), and exchanging group from the original biological into its biosimilar (INF/CT-P13). The exchange group showed non-inferiority to the ongoing treatment with INF on efficacy and safety for all diseases investigated. However, there was not enough statistical power to demonstrate the non-inferiority for each disease studied. This research was financed by the Norwegian Government.

### Efficacy

Different measurements were used according to clinical population studied. Authors define two main types of variables: a) *categorical (state)*: percentage of patients with a specified condition (deterioration or remission) based on clinical scales, and b) *numerical (change)*: any variation in the score of clinical scales at the end of the follow-up with respect to the baseline (exchange time).

#### Deterioriation During Follow-Up

This was the primary outcome for all patients based on specific clinical scales for each of the six diseases studied. In the ITT analysis, frequency of deterioration in all diseases were 22.4% in the INF/INF group, and a 26.3% in the INF/CTP-13 group (p=0.3259). Although the frequency of decline of the six diseases was defined, there was not enough statistical power to test non-inferiority of each disease; thus, we only report the frequencies for exploratory purposes (**Table 3C**).

**Table 3C:** Frequency of deterioration and remission during follow-up in patients with Crohn’s disease, ulcerative colitis, spondylarthritis, rheumatoid arthritis, psoriatic arthritis, and chronic plaque psoriasis.

				Worsening during follow-up							Remission during follow-up
Author (Year)	Time	Groups	Patients allocated	All diseases	Rheumatoid arthritis	Psoriatic arthritis	Psoriasis	Spondylarthritis	Crohn’s Disease	Ulcerative colitis	All Diseases
Jorgensen et al. (2017)	*Start:* Week 0	INF/INF	241	54 (22.4%)	11(28.2%)	7 (50%)	2 (11.1%)	17 (37.8%)	14 (17.9%)	3 (6.4%)	145 (60.2%)
	*End:* Week 52	INF/CT-P13	240	63 (26.3%)	*10 (26.3%)*	*8 (50.0%)*	*2 (11.8%)*	*14 (30.4%)*	*24 (31.2%)*	*5 (10.9%)*	*146 (60.8%)*
		*Estimated p-value*		p=0.3259	NE	NE	NE	NE	NE	NE	p=0.8810

NE, Not estimated due to the low statistical power.

#### Remission During Follow-Up

Approximately 60% of patients in each group achieved remission (p=0.8810) (**Table 3C**). There was not enough statistical power to evaluate the non-inferiority for each disease.

#### Quality of Life: SF36 and EQ5D

Health-related quality of life (QoL) for all diseases were assessed using SF36 and EQ5D; two validated and widely used instruments. In the first group, statistically significant differences during the follow-up period regarding physical limitations (p=0.0069) and emotional limitations were found (p=0.026); with a greater average of deterioration (decrease in score) in the maintenance group (-0.4) and exchange group (-1.1). There were not statistical differences in the others components. Meanwhile, there were different changes on the clinical global impressions scale of EQ5D in both groups (p=0.999) (**Table 3D**).

**Table 3D:** Quality of life in patients with Crohn’s disease, ulcerative colitis, spondylarthritis, rheumatoid arthritis, psoriatic arthritis, and chronic plaque psoriasis (SF36 and EQ5D).

Author (Year)	Time	Groups (patients allocated)	SF-36 FF	SF-36 LRF	SF-36 Pain	SF-36 SG	SF-36 BE	SF-36 LRE	SF-36 FS	SF-36 EF	SF-36 RCF	SF-36 RCM	EQ 5D
Jørgensen et al. (2017)	*Exchange:* Week 0	INF/INF (n=241)	*Baseline:* 50.6 (11.3) *End:* –1.2 (7.0)	*Baseline:* 45.6 (11.6) *End:* –1.1 (11.2)	*Baseline:* 47.2 (8.5) *End:* –0.7 (7.3)	*Baseline:* 43.5 (10.2) *End:* –1.1 (7.3)	*Baseline:* 50.0 (9.8) *End:* –1.3 (7.8)	*Baseline:* 48.8 (10.8) *End:* –0.5 (12.2)	*Baseline:* 48.0 (10.5) *End:* –0.2 (9.4)	*Baseline:* 47.1 (10.4) *End:*–1.9 (8.5)	*Baseline:* 46.4 (10.1) *End:* –1.2 (6.9)	*Baseline:* 49.1 (10.7) *End:* –0.7 (8.9)	*Baseline:* 0.8 (0.2) *End:* 0.0 (0.2)
	*End:* Week 52	INF/CT-P13 (n=240)	*Baseline:* 50.5 (10.9) *End:* 0 (6.3)	*Baseline:* 46.9 (11.3) *End:* –0.4 (9.4)	*Baseline:* 47.8 (9.5) *End:* –0.5 (7.7)	*Baseline:* 44.5 (10.2) *End:* –1.1 (7.1)	*Baseline:* 50.9 (8.9) *End:* –0.7 (7.8)	*Baseline:* 50.0 (10.4) *End:* –2.4 (10.5)	*Baseline:* 48.6 (9.5) *End:* –0.6 (10.4)	*Baseline:* 46.9 (10.2) *End:* 0.5 (8.3)	*Baseline:* 46.8 (10.3) *End:* 0.2 (6.6)	*Baseline:* 50.3 (9.3) *End:* –1.3 (8.9)	*Baseline:* 0.8 (0.2) *End:* 0.0 (0.2)
		*Estimated p-value*	0.103	0.0069	0.4096	0.6677	0.999	0.026	0.1183	0.7129	0.4921	*0.999*	*0.999*

SF-36, 36-Item Short Form Health Survey; FF, Physical functioning; LRF, Limitation of physical roles; SG, General Health; BE, Emotional wellbeing; LRE, Limitation of emotional roles; FS, Social Functioning; EF, Energy or Fatigue; RCF, Physical component summary; RCM, Mental component summary; Baseline, exchanging time; End, Conclusion of follow-up.

### Safety

There were no statistically significant differences between patients of two groups in safety variables.

#### Adverse Events

Frequencies of serious AE were 10% in maintenance patients and 9% in exchanging group. Discontinuation due to AE was 4% and 3%, respectively.

#### Immunogenecity

Frequency of patients with post transition ADA were: 7% (INF/INF) and 8% (INF/CTP-13) (**Table 3E**).

**Table 3E:** Safety outcomes in all primary studies included.

Author (Year)	Conditions	Exchanging Time	Intervention Groups	Patients allocated	Immunogenicity (ADA)	Patients with adverse events (post exchange)
Smolen et al.(2018)	Rheumatoid arthritis	Exchange: Week 54 End: Week 78	INF/INF	101	*Post-transition:* *14.9%*	Any AE: 36(35.6%)Serious AE: 3 (3%)Discontinuation due to AE: 1 (1%)
			SB2/SB2	201	*Post-transition:* *14.1%*	Any AE:81(40.3%)Serious AE: 7(3.5%)Discontinuation due to AE: 3 (1.5%)
			INF/SB2	94	*Post-transition:* *14.6%*	Any AE: 34(36. 2%)Serious AE: 6 (6.4%)Discontinuation due to AE: 3 (3.2%)
		*Estimated p-value*			*p=0.98*	
Jorgensen et al. (2017)*	Crohn's disease. Ulcerative colitis.Rheumatoid arthritis. Spondylarthritis. Psoriatic arthritis. Chronic plaque psoriasis	Exchange: Week 0 End: Week 52	INF/INF	241	*Post-transition:* 17 (7.1%)	Any AE: 168 (70%)Serious AE: 24 (10%)Discontinuation due to AE: 9(4%)
			INF/CT-P13	*240*	*Post-transition:* 19 (7.9%)	Any AE: 164 (68%)Serious AE: 21(9%)Discontinuation due to AE: 8(3%)
		*Estimated p-value*			0.911	
Tanaka et al.(2017)*	Rheumatoid arthritis	Exchange: Week 54 End: Week 167	CT-P13/CT-P13	38	*Post-transition:* *4 (10.6%)*	Any AE: 34(89. 5%)Serious AE: 2(5.3%)Discontinuation due to AE: 4(10.5%)
			INF/CT-P13	33	*Post-transition:* *4 (12.1%)*	Any AE: 29 (87.9%)Serious AE: 4 (12.1%)Discontinuation due to AE: 8 (24.2%)
		*Estimated p-value*			*0.901*	
Yoo et al.(2017)	Rheumatoid arthritis	Exchange: Week 54 End: Week 102	CT-P13/CT-P13	158	*Post-transition:* 64(40.5%)	Any AE: 85 (53.8%) Serious AE: 12(7.5%)Discontinuation due to AE: 16 (10.1%)
			INF/CT-P13	144	*Post-transition:* 64 (44.4%)	Any AE: 77 (53.5%) Serious AE: 13(9.0%)Discontinuation due to AE: 8 (5.6%)
		*Estimated p-value*			0.48	
Park et al.(2017)	Ankylosing spondylitis	Exchange: Week 54 End: Week 102	CT-P13/CT-P13	88	*Post-transition:* 21 (23.3%)	Any AE: 44 (50%) Serious AE: 4 (4.5%)Discontinuation due to AE: 3 (3.3%)
			INF/CT-P13	86	*Post-transition:* 23 (27.4%)	Any AE: 60 (69.7%)Serious AE: 4 (4.6%)Discontinuation due to AE: 4 (4.6%)
		*Estimated p-value*			0.60	

ADA, Anti-drug antibody; NR, Not reported; EA, Adverse Events. Primary outcome was cumulative incidence of AE.

Tanaka et al., 2017. **“Safety and Efficacy of CT-P13 in Japanese Patients with Rheumatoid Arthritis in an Extension Phase or after Switching from Infliximab.” Modern Rheumatology 27 (2): 237–45** (Tanaka et al. 2017).

This open label study, RA patients were randomized in two arms: INF/CTP-13 and CTP-13/CTP-13. There were no statistical differences in efficacy and safety assessed by clinical scales.

### Efficacy

#### ACR20, ACR50 and ACR70

No differences were found in frequency of patients who improved in the three categories proposed by the American College of Rheumatology: ACR20%, ACR50% and ACR70%. In CTP-13/CTP-13 (78.4%, 70.3% and 54.1%) and INF/CTP-13 (62.5%, 53.1% and 40.6%), respectively (**Table 3A**).

#### DAS28

There were also no differences in the average scores at the end of the follow-up between maintenance group (2.78) and exchange group (2.03) (p=0.612) (**Table 3A**).

#### EULAR

Frequency of good or moderate response after the follow-up period did not show significant statistical difference between the two groups: 83% in maintenance patients and 68.8% in exchanging people (**Table 3A**).

### Safety

#### Adverse Events

In maintenance group, 5.3% of patients had serious AE and 10.5% discontinuing the prescription due to AE. Meanwhile, in CTP-13 exchanging group participants had 12.1% of serious AE and 24.2% discontinued the treatment due to AE. These differences were not statistically significant (**Table 3E**).

#### Immunogenicity

In post-transition stage, frequency of patients with ADA were 10.6% in maintenance group and 12.1% in exchanging group. (p=0.901) (**Table 3E**).

**“Efficacy and Safety of CT-P13 (Biosimilar Infliximab) in Patients with Rheumatoid Arthritis: Comparison between Switching from Reference Infliximab to CT-P13 and Continuing CT-P13 in the PLANETRA Extension Study.” Annals of the Rheumatic Diseases 76 (2): 355–63** (Yoo et al., 2017).

Authors compared two groups of Rheumatoid arthritis patients: maintenance (CTP-13/CTP-13) and exchanging (INF/CTP13). Efficacy, tolerability and safety observed were non different between groups.

#### ACR20, ACR50 and ACR70

Frequencies of 20%, 50% and 70% responses according to the ACR criteria were: 74.1%, 49.4% and 24.7% in CTP-13/CTP-13, and 77.1%, 54.2% and 26.4% in INF/CTP-13 group. There was no evidence of statistically significant differences between groups (**Table 3A**).

#### DAS28

The average final scores were not statistically different between maintenance (2.40) and exchanging (2.48) groups (**Table 3A**).

#### EULAR

Frequency of patients with a good or moderate criterion according to EULAR were 80.9% and 81% in maintenance and exchanging arms, respectively. There were no statistical differences between groups (p=0.669) (**Table 3A**).

### Safety

#### Adverse Events

In maintenance group, 7.5% of patients had serious AE, and 10% discontinued their treatment due to AE. These frequencies did not differ than exchanging group (9% and 5.6%, respectively) (**Table 3E**).

Park et al., 2017. **“Efficacy and Safety of Switching from Reference Infliximab to CT-P13 Compared with Maintenance of CT-P13 in Ankylosing Spondylitis: 102-Week Data from the PLANETAS Extension Study” Annals of the Rheumatic Diseases 76 (2): 346–54** (Park et al., 2017).

This trial was carried out in patients with ankylosing spondylitis. Participants were randomized in two groups: maintenance (CTP-13/CTP-13) and exchanging (INF/CTP-13). No statistically significant differences were observed between group in terms of efficacy or safety.

### Efficacy

#### Assessment of Spondylarthritis international Society (ASAS20, ASAS40 and ASAS PR)

Non statistical differences were found between groups according ASAS measurements for 20%, 40% and partial remission of disease: in maintenance (80.7%, 63.9% and 19.3%) and exchanging (76.9%, 61.5% and 23.1%) patients (**Table 3B**).

#### Bath Ankylosing Spondylitis Disease Activity Index (BASDAI), Bath Ankylosing Spondylitis Functional Index (BASFI), Ankylosing Spondylitis Disease Activity Score (ASDAS) and Bath Ankylosing Spondylitis Metrology Index (BASMI)

Authors reported - using graphical methods - non differences in average at the end of follow-up between maintenance and exchanging groups: BASDAI (3.19 vs. 3.23), BASFI (3.1 vs. 3.25), ASDAS (1.86 vs. 1.97), and BASMI (2.4 vs. 2.6) (**Table 3B**).

### Safety

#### Adverse Events

In maintenance group, 4.5% and 3.3% of allocated patients had serious AE and discontinued treatment due to AE, respectively. Meanwhile, in exchanging group frequencies were 4.6% in both measures. There are no evidence of statistical differences between arms (**Table 3E**).

#### Immunogenicity

In post-exchange period, there were non statistical differences in proportion of patients with ADAs between groups. Authors reported 23.3% in CTP-13/CTP-13 and 27.4% in INF/CTP-13 groups (**Table 3E**).

### Preliminary Financial Analysis

First, we present the estimate of annual costs per patient based on price for each vial offered by each provider, S/2040.00 (S/: Peruvian soles) for BRP and S/857 for BBP; and number of vials required per patient (annual average). This implies annual savings around S/24843 per-patient with biosimilar. Secondly, we estimated the cost differences based on annual requirement of Infliximab from EsSalud (6460 vials); thus, the biosimilar introduction could produce savings around S/7´642,780.00 (1 USD: S/3.30) (**Table 4**).

**Table 4:** Preliminary financial analysis about the cost related to treatment with infliximab an its biosimilar in EsSalud (1USD = S/3.30).

Biological Product	Supplier	Estimation of annual costs per patient				EsSalud Annual Purchase	
		Unit cost per vial*	Average requirement per application per patient **	Frequency of annual application***	Average annual cost per patient	Annual requirement****	Total annual cost
Infliximab Original	*Johnson& Johnson*	*S/2,040.00*	*3 vials*	*7 times*	*S/42,840.00*	*6460 vials*	S/13,178,400.00
Biosimilar	*AC Farma*	*S/857.00*			*S/17,997.00*		S/5,536,220.00
Difference		*-S/1,183.00*			*-S/24,843.00*		-S/7,642,780.00

*Based on what was sold by the suppliers in the last purchase of the biological product registered in the SEACE platform for a vial of infliximab of 100 mg.

**Estimated for 60 kg person on average at a dose of 5mg / kg.

***The application is every 8 weeks on average; the annual estimate has been rounded.

****The total requirement corresponds to what was requested by EsSalud for the year 2018.

### Technical Discussion With Medical Doctors

In first meeting, we received questions and feedback from rheumatologists, dermatologists and gastroenterologists. In second meeting, we discussed those questions an related legal aspects, and we also defined scope and limitations of analysis performed. At the last meeting, we presented and received the approbation of final technical document. The main concerns expressed by doctors were not being able to conclude for each disease separately, nocebo effect and using of generic questionnaires to assess the quality of life. We address them in the discussion.

## Discussion

Our findings reflect the best primary evidence available related to continuation with a biological biosimilar drug in patients that receive Infliximab -as biological reference drug - in conditions approved by the Peruvian Social Security. While only two of the studies respond directly to PICOS question using Infliximab as original maintenance drug, we included all controlled trials that evaluated interchangeability from the original Infliximab into its biosimilar. All primary studies did not find statistical and clinical differences between maintenance and exchanging groups in efficacy and safety profiles. Moreover, in comparison with infliximab, the use of its biosimilar produce a substantial savings in EsSalud purchasing budget. In addition, both analyzes were discussed and accepted by rheumatologists, dermatologists and gastroenterologists working in EsSalud. In this sense, our manuscript is an integrated technical piece, which embraces scientific evidence, institutional budget and clinical experience about infliximab interchangeability in the complexity of Peruvian Health System, where EsSalud is one of the foremost public institutions with assurance, provision and health care functions (Sánchez-Moreno, 2014; Mezones-Holguin et al., 2019). Consequently, we described a mixed methodological approach to inform making-decisions with the best available evidence in low and middle-income countries context.

In the academic realm, other systematic reviews have addressed the interchangeability from original into biosimilar drugs. First, *Chingcuanco* et al., performed a SR in Pubmed, EMBASE, CENTRAL and LILACS until April-2016; they concluded that there is primary evidence that supports interchangeability from Biological reference products to biosimilar drugs in TNF-α family (Chingcuanco et al., 2016). Second, *Cohen et al*., carried out a SR including interventional and observational clinical studies in MEDLINE and EMBASE until June-2017; in this review the risk of events related to immunogenicity and declination of efficacy did not change after exchanging from original to biosimilar (Cohen et al., 2018). Third, *McKinnon et al.*, published an SR performed in Pubmed, EMBASE and Cochrane Library until June-2017 to evaluate the efficacy and safety of biosimilar interchangeability. There were still gaps to determining safety and efficacy of interchangeability of biosimilar was their conclusion, although they did not provide any specific conclusion about infliximab (McKinnon et al., 2018). Fourth, *Feagan et. al*, recently published a SR, they searched until January-2018 in Medline for articles and EMBASE for abstract congress. Six RCT and 64 observational studies were included. The authors described that “the evidence revealed no clinically important efficacy or safety signals associated with switching” (Feagan et al., 2019). Consequently, none of those synthesis studies reported differences in efficacy or safety between maintenance or exchanging into biosimilar, however there are different opinions in recommending the continuation with biosimilar drug.

In technical meetings with specialists, some concerns were exposed. The first was the inability to make specific comparisons for each disease separately- due to low statistical power- specifically in the clinical trial financed by the Government of Norway conducted in patients with rheumatoid arthritis, severe psoriasis, ulcerative colitis, Crohn’s disease and spondylarthrosis. (Jørgensen et al., 2017). Although the authors performed subgroup analyses for each disease and reported findings with no statistically significant differences for several specific outcomes, their results were exploratory and could be affected by selection bias (Assmann et al., 2000; Brookes et al., 2004). However, valid conclusions were obtained for all diseases studied. In this regard, the authors report three main outcomes of efficacy (worsening of the disease, remission of the disease, and quality of life) and two safety outcomes (adverse effects and immunogenicity) for all diseases included. They observed non statistical differences between groups with adequate statistical power. (Jørgensen et al., 2017). Therefore, the first two outcomes of efficacy were the total percentage of patients who had worsening or remission of the disease in each group. Definition of state was based on medical evaluation supported by validated and accepted specific clinical scales for each disease (Jørgensen et al., 2017).

On the other hand, the use of generic questionnaires to assess quality of life (QoL) across the diseases was the second concern. *Jorgensen* et al. did not find differences in the SF-36 and EQ5D between the maintenance and switched groups (Jørgensen et al., 2017). QoL is widely recognized as a valid outcome in clinical studies and as basis for calculating utility measurements (Drummond et al., 2005; Bottomley et al., 2018). SF-36 and EQ5D can be used in any health conditions; both scales has been used by National Institute of Clinical Excellence (NICE) to assess efficacy of interventions in several diseases, including: rheumatological, dermatological and gastroenterological (Longworth et al., 2014). It is noteworthy that these two generic indices allow us to estimate utilities measures - as Quality of life adjusted life years (QALY) - for comparing across different health conditions (Rabarison et al., 2015). In the following two paragraphs we provide a succinct description of those tools in relation to diseases evaluated.

The SF-36 is widely used worldwide and it has evidence of validity and reliability in Peru (Salazar and Bernabé, 2015). A series of SR show that this tool has adequate psychometric properties in patients with RA (Matcham et al., 2014), it allows to quantify worsening psoriasis in clinical trials (Ali et al., 2017) and it is a valid outcome in patients with psoriatic arthritis that receive biological drugs (Druyts et al., 2017). In addition, for inflammatory bowel disease, SR described that SF-36 is useful to assess the quality of life and it provides evidence of variations in stages of activity and inactivity of the disease (Knowles et al., 2018) and a Cochrane systematic review describe that SF-36 is a valid and reliable instrument to evaluate the effect of biological therapy. (LeBlanc et al., 2015). Moreover, other SRs argue that this questionnaire has psychometric validity (Yarlas et al., 2018a) and serves to estimate the burden of disease in patients with ulcerative colitis (Yarlas et al., 2018b). Also, SF-36 is useful to assess QoL in patients with ankylosing spondylitis (Yang et al., 2016).

The EQ5D is a tool developed by EUROQOL that, with appropriate contextualization, serves as the basis for the calculation of QALYs (Brazier et al., 2017; Dakin et al., 2018). There is a Peruvian version of EQ5D (Szende et al., 2007; Brooks et al., 2013). In patients with rheumatoid arthritis its use has been described in the estimation of utility measures (Boyadzieva et al., 2018), clinical practice (Hiligsmann et al., 2018), also it has a good correlation with disease activity (Skacelova et al., 2017). EQ-5D is a valid and reliable instrument in the assessment of worsening in clinical trials conducted in patients with psoriasis. (Ali et al., 2017). Also, it is used in patients with plaque psoriasis, psoriatic arthritis (Longworth et al., 2014; Yang et al., 2016) and multicenter studies of skin diseases (Balieva et al., 2017). A Cochrane SR described that EQ-5D was adequate in the evaluation of the effectiveness of treatment with biological drugs in patients with inflammatory bowel disease (LeBlanc et al., 2015). Likewise, EQ-5D is an adequate tool to measure quality of life in ankylosing spondylitis patients (Boonen et al., 2007) and high correlation with specific scales of the disease has been observed (Mlcoch et al., 2017).

Similarly, safety is a highly important outcome studied. We defined immunogenicity and adverse events as main safety results. Immunogenicity is a relevant marker in the biotherapeutics research, since the production of anti-drug antibodies is clearly associated with therapeutic failure and side effects of protein drugs (Ingrasciotta et al., 2018). Also, immunogenicity of Infliximab biosimilar can be extrapolated to the different diseases treated (Ben-Horin et al., 2015). Moreover, the adverse events, especially the serious ones, are valid safety outcomes for a biological drug in the context of clinical trials (Tridente, 2013). Subsequently, we incorporated two main safety measures in the biosimilars arena.

Our study has potential limitations. First, we did not include unpublished studies from the gray literature (reports, conference proceedings, doctoral theses/dissertations, etc.), which may imply a selection bias. But, critical appraisal of the evidence is essential for developing a SR, since, although the findings can be made known, we cannot evaluate their quality, which has repercussions on the validity and reliability of a synthesis study (Bolaños-Díaz et al., 2011). Second, non-inclusion of observational studies could be a selection bias source, even more when the academy recognizes them as a valid source of clinical evidence (Greenfield, 2017; Corrao and Cantarutti, 2018). Nevertheless, our manuscript is circumscribed in a specific decision-making environment, where there is an institutional regulatory framework. In EsSalud, IETSI has defined that– based on internal validity criterion- randomized clinical trials are the main source of evidence to inform making-decisions; in addition, the overall results of SRs - that included observational studies - did not differ from RCT findings and provide consistency to our results. Third, we did not carry out a quantitative synthesis of the studies, due to the enormous clinical and methodological heterogeneity, but in this situation performing meta-analysis is not advisable (Melsen et al., 2014). Fourth, we have not considered drop-out rates and nocebo effect, which could potentially exist in switched patients (from original into biosimilar) (Kristensen et al., 2018; Odinet et al., 2018); education provided prior to switch -among other interventions - is a valuable tool that can greatly help overcome this effect (Pouillon et al., 2019). Fifth, in the financial analysis, we do not have the official information of short-term patients and long-term chronic patients in each disease approved; however, our estimation is valid since it was based on absolute institutional annual requirement of infliximab. Sixth, we did not have a national representative sample of physicians; however, participants were working in the main healthcare networks of EsSalud.

Beyond the limitations and based on cost-opportunity as a legitimate principle of collective health, our findings support the use of a biosimilar to continue the treatment in patients receiving infliximab in EsSalud. Therefore, biosimilar constitute a valid therapeutic alternative for the management of medical conditions approved in EsSalud. Access to biological drugs is a struggle for health care systems, especially in low and middle-income economies, where a key aspect is the price of these innovative medicines, which leads to a significant economic exertion from Governments and their public budgets. In this sense, infliximab biosimilars are an alternative that could be efficient in the Peruvian Social Security context.

## Author Contributions

EM-H, FF, MC-R, PB-P, GS-F, RG-C, and JC-C participated in the conception of the study and the research question. EM-H, LL-S, and LH-S designed the systematic review, developed the search strategy, and selected the articles. EM-H, FF, PB-P, GS-F, MC-R, JC-C, and RG-C defined and discussed the outcomes of interest. EM-H, LL-S, and LH-S performed the extraction and preliminary drafting of the results. EM-H, FF, PB-P, and MC-R carried out the cost estimates. EM-H, FF, PB-P, GS-F, MC-R, JC-C, and RG-C reviewed the results and delineated the discussion. EM-H, LL-S, and LH-S made the first version of the article. EM-H, FF, MC-R, PB-P, LH-S, LL-S, JC-C, GS-F, and RG-C made substantial contributions to the manuscript. All authors agreed with the published version of the article and assume responsibility for its content.

## Conflict of Interest

RG-C has received funding for travel, accommodation, or expenses for congresses by Pfizer and Novartis, and has also received funding or contracts from Pfizer in health research projects. M-H has carried out academic activities financed indirectly by Jansen, a Johnson & Johnson company.

The remaining authors declare that the research was conducted in the absence of any commercial or financial relationships that could be construed as a potential conflict of interest.
