# Supplementary material for: Efficacy and Safety in the Continued Treatment With a Biosimilar Drug in Patients Receiving Infliximab: A Systematic Review in the Context of Decision-Making From a Latin-American Country
Source: Front Pharmacol. 2019 Nov 15;10:1010. doi: 10.3389/fphar.2019.01010 (PMC6874174; doi:10.3389/fphar.2019.01010)
Supplement: Supplementary file 1 [file Table_1.docx]

**Annex A.** Pubmed-MEDLINE

| Terms | # |
| --- | --- |
| "Arthritis, Rheumatoid"[Mesh] OR "Rheumatoid Arthritis" OR Rheumatoid OR arthritis OR polyarthritis OR polyarthritides | 1 |
| "Psoriasis"[Mesh] OR psorias* OR psoriat* OR "Pustulosis of Palms and Soles" OR "Pustulosis Palmaris et Plantaris" OR "Palmoplantaris Pustulosis" OR "Pustular Psoriasis of Palms and Soles" OR "pustular palmoplantar psoriasis" OR "Arthritis, Psoriatic"[Mesh] OR "Arthritic Psoriasis" OR "Psoriatic Arthritis" OR "Psoriasis Arthropathica" OR "Psoriatic Arthropathy" OR "plaque psoriasis" OR "psoriasis vulgaris" OR "severe psoriasis" | 2 |
| "Colitis, Ulcerative"[Mesh] OR "Ulcerative Colitis" OR "Idiopathic Proctocolitis" OR "Colitis Gravis" | 3 |
| "Crohn Disease"[Mesh] OR "Crohn Disease" OR "Crohn's Enteritis" OR "Regional Enteritis" OR "Crohn's Disease" OR "Inflammatory Bowel Disease 1" OR "Granulomatous Enteritis" OR Ileocolitis OR "Granulomatous Colitis" OR "Terminal Ileitis" OR "Regional Ileitides" OR "Regional Ileitis" | 4 |
| #1 OR #2 OR #3 OR #4 | 5 |
| "Infliximab"[Mesh] OR Infliximab OR "MAb cA2" OR "Antibodies, Monoclonal"[Mesh] OR "Monoclonal Antibod*" OR "Monoclonal Antibody cA2" OR Remicade OR "Anti-TNF-α" | 6 |
| #5 AND #6 | 7 |
| "Biosimilar Pharmaceuticals"[Mesh] OR "Biosimilar Pharmaceuticals" OR biosimilar* OR "subsequent entry biologics"OR "subsequent entry biologic"OR "similar biotherapeutic product*" OR "similar biotherapeutic*" OR "similar biological medicinal product*" OR "similar biologicals" OR "similar biological" OR Biogeneric* OR "follow-on biologic" OR "follow-on biologics" OR TuNEX OR HD203 OR SB2 OR BOW015 OR SB4 OR ZRC-3197 OR GP2015 OR GP2017 OR "ABP 501" OR "CT-P13"[Supplementary Concept] OR CT-P13 OR Inflextra OR Inflectra OR Remsima | 8 |
| "Drug Substitution"[Mesh] OR "Drug Substitution*" OR substitut* OR "Drug Switching" OR switch* OR interchange* OR equivale* OR equianalg* OR equipotency OR comparability OR conversion OR rotation OR dose ratio | 9 |
| #8 AND #9 | 10 |
| #7 AND #10 | 11 |

**Annex B.** Web of Science (WOS)

| Terms | # |
| --- | --- |
| TS=("Rheumatoid Arthritis" OR Rheumatoid OR arthritis OR polyarthritis OR polyarthritides) | 1 |
| TS=(Psoriasis OR psorias* OR psoriat* OR "Pustulosis of Palms and Soles" OR "Pustulosis Palmaris et Plantaris" OR "Palmoplantaris Pustulosis" OR "Pustular Psoriasis of Palms and Soles" OR "pustular palmoplantar psoriasis" OR "Arthritic Psoriasis" OR "Psoriatic Arthritis" OR "Psoriasis Arthropathica" OR "Psoriatic Arthropathy" OR "plaque psoriasis" OR "psoriasis vulgaris" OR "severe psoriasis") | 2 |
| TS=("Ulcerative Colitis" OR "Idiopathic Proctocolitis" OR "Colitis Gravis") | 3 |
| TS=("Crohn Disease" OR "Crohn's Enteritis" OR "Regional Enteritis" OR "Crohn's Disease" OR "Inflammatory Bowel Disease 1" OR "Granulomatous Enteritis" OR Ileocolitis OR "Granulomatous Colitis" OR "Terminal Ileitis" OR "Regional Ileitides" OR "Regional Ileitis") | 4 |
| #1 OR #2 OR #3 OR #4 | 5 |
| TS=(Infliximab OR "MAb cA2" OR "Monoclonal Antibod*" OR "Monoclonal Antibody cA2" OR Remicade OR "Anti-TNF-α") | 6 |
| #5 AND #6 | 7 |
| TS=("Biosimilar Pharmaceuticals" OR biosimilar* OR "subsequent entry biologics"OR "subsequent entry biologic"OR "similar biotherapeutic product*" OR "similar biotherapeutic*" OR "similar biological medicinal product*" OR "similar biologicals" OR "similar biological" OR Biogeneric* OR "follow-on biologic" OR "follow-on biologics" OR TuNEX OR HD203 OR SB2 OR BOW015 OR SB4 OR ZRC-3197 OR GP2015 OR GP2017 OR "ABP 501" OR CT-P13 OR Inflextra OR Inflectra OR Remsima) | 8 |
| TS=("Drug Substitution" OR "Drug Substitution*" OR substitut* OR "Drug Switching" OR switch* OR interchange* OR equivale* OR equianalg* OR equipotency OR comparability OR conversion OR rotation OR (dose ratio)) | 9 |
| #8 AND #9 | 10 |
| #7 AND #10 | 11 |

**Annex C.** Excerpta Medica (EMBASE)

| Terms | # |
| --- | --- |
| 'rheumatoid arthritis'/mj OR 'juvenile rheumatoid arthritis' OR 'rheumatic disease' OR 'reactive arthritis' OR 'polyarthritis' OR 'monarthritis' | 1 |
| 'psoriasis'/mj OR 'pustulosis palmoplantaris' OR 'psoriatic arthritis' OR 'erythrodermic psoriasis' OR 'guttate psoriasis' OR 'psoriasis vulgaris' OR 'pustular psoriasis' OR 'psoriatic arthritis' | 2 |
| 'ulcerative colitis'/mj | 3 |
| 'Crohn disease'/mj OR 'colon Crohn disease' OR 'inflammatory bowel disease' OR 'enteritis' OR 'ileocolitis' OR 'granulomatous colitis' | 4 |
| #1 OR #2 OR #3 OR #4 | 5 |
| 'infliximab'/mj OR 'monoclonal antibody' OR 'monoclonal antibody ca2' OR 'tumor necrosis factor antibody' | 6 |
| #5 AND #6 | 7 |
| 'biosimilar agent'/mj OR 'similar biotherapeutic product' OR 'biological product' OR 'biologic factors and agents acting on the immune system' OR 'biological therapy' OR 'adalimumab' OR 'ct p13' OR 'Sb2' | 8 |
| 'drug substitution'/mj OR 'substitution therapy' OR 'switch' OR 'equivalence trial' OR 'comparative effectiveness' OR 'dose ratio' | 9 |
| #8 AND #9 | 10 |
| #7 AND #10 | 11 |

**Annex D.** SCOPUS

| Terms | # |
| --- | --- |
| "Rheumatoid Arthritis" OR Rheumatoid OR arthritis OR polyarthritis OR polyarthritides | 1 |
| Psoriasis OR psorias* OR psoriat* OR "Pustulosis of Palms and Soles" OR "Pustulosis Palmaris et Plantaris" OR "Palmoplantaris Pustulosis" OR "Pustular Psoriasis of Palms and Soles" OR "pustular palmoplantar psoriasis" OR "Arthritic Psoriasis" OR "Psoriatic Arthritis" OR "Psoriasis Arthropathica" OR "Psoriatic Arthropathy" OR "plaque psoriasis" OR "psoriasis vulgaris" OR "severe psoriasis" | 2 |
| "Ulcerative Colitis" OR "Idiopathic Proctocolitis" OR "Colitis Gravis" | 3 |
| "Crohn Disease" OR "Crohn's Enteritis" OR "Regional Enteritis" OR "Crohn's Disease" OR "Inflammatory Bowel Disease 1" OR "Granulomatous Enteritis" OR Ileocolitis OR "Granulomatous Colitis" OR "Terminal Ileitis" OR "Regional Ileitides" OR "Regional Ileitis" | 4 |
| #1 OR #2 OR #3 OR #4 | 5 |
| Infliximab OR "MAb cA2" OR "Monoclonal Antibod*" OR "Monoclonal Antibody cA2" OR Remicade OR "Anti-TNF-α" | 6 |
| #5 AND #6 | 7 |
| "Biosimilar Pharmaceuticals" OR biosimilar* OR "subsequent entry biologics"OR "subsequent entry biologic"OR "similar biotherapeutic product*" OR "similar biotherapeutic*" OR "similar biological medicinal product*" OR "similar biologicals" OR "similar biological" OR Biogeneric* OR "follow-on biologic" OR "follow-on biologics" OR TuNEX OR HD203 OR SB2 OR BOW015 OR SB4 OR ZRC-3197 OR GP2015 OR GP2017 OR "ABP 501" OR CT-P13 OR Inflextra OR Inflectra OR Remsima | 8 |
| "Drug Substitution" OR "Drug Substitution*" OR substitut* OR "Drug Switching" OR switch* OR interchange* OR equivale* OR equianalg* OR equipotency OR comparability OR conversion OR rotation OR (dose ratio) | 9 |
| #8 AND #9 | 10 |
| #7 AND #10 | 11 |

**Annex E.** Cochrane Electronic Register of Controlled Trials (CENTRAL)

| **Terms** | **#** |
| --- | --- |
| “Arthritis, Rheumatoid” | 1 |
| Psoriasis | 2 |
| “Colitis, Ulcerative” | 3 |
| “Crohn Disease” | 4 |
| #1 OR #2 OR #3 OR #4 | 5 |
| Infliximab | 6 |
| #5 AND #6 | 7 |
| “Biosimilar Pharmaceuticals” | 8 |
| “Drug Substitution” | 9 |
| #8 OR #9 | 10 |
| #7 AND #10 | 11 |

**Annex F.** Traslating Research into Practice (TRIPDATABASE)

| Términos | # |
| --- | --- |
| Infliximab OR "MAb cA2" OR "Monoclonal Antibod*" OR "Monoclonal Antibody cA2" OR Remicade OR "Anti-TNF-α" | 1 |
| "Biosimilar Pharmaceuticals" OR biosimilar* OR "subsequent entry biologics"OR "subsequent entry biologic"OR "similar biotherapeutic product*" OR "similar biotherapeutic*" OR "similar biological medicinal product*" OR "similar biologicals" OR "similar biological" OR Biogeneric* OR "follow-on biologic" OR "follow-on biologics" OR TuNEX OR HD203 OR SB2 OR BOW015 OR SB4 OR ZRC-3197 OR GP2015 OR GP2017 OR "ABP 501" OR CT-P13 OR Inflextra OR Inflectra OR Remsima | 2 |
| "Drug Substitution" OR "Drug Substitution*" OR substitut* OR "Drug Switching" OR switch* OR interchange* OR equivale* OR equianalg* OR equipotency OR comparability OR conversion OR rotation OR (dose ratio) | 3 |
| #2 AND #3 | 4 |
| #1 AND #4 | 5 |

**Annex G.** National Institute for Health and Care Excellence (NICE)

| **Términos** | **#** |
| --- | --- |
| Infliximab | 1 |
| Biosimilar | 2 |
| #1 AND #2 | 3 |

**Annex H.** The Agency for Healthcare Research and Quality (AHRQ)

| **Términos** | **#** |
| --- | --- |
| Infliximab | 1 |
| switch OR interchange OR substitute | 2 |
| #1 AND #2 | 3 |

**Anexo I.** The Scottish Medicines Consortium (SMC)

| **Términos** | **#** |
| --- | --- |
| Infliximab | 1 |

**Anexo J.** The Canadian Agency for Drugs and Technologies in Health (CADTH)

| **Términos** | **#** |
| --- | --- |
| Infliximab | 1 |
| "Biosimilar Pharmaceuticals" | 2 |
| #1 AND #2 | 3 |

**Annex K.** The Health Systems Evidence (HSE)

| **Términos** | **#** |
| --- | --- |
| Infliximab OR "MAb cA2" OR "Monoclonal Antibody*" OR "Monoclonal Antibody cA2" OR Remicade OR "Anti-TNF-α" | 1 |

**Annex L**. International Clinical Trials Registry Platform (ICTRP) - World Health Organization (http://www.who.int/ictrp/en)

| **Términos** | **#** |
| --- | --- |
| Infliximab OR "MAb cA2" OR "Monoclonal Antibod*" OR "Monoclonal Antibody cA2" OR Remicade OR "Anti-TNF-α" | 1 |
| switch OR interchange OR substitute | 2 |
| #1 AND #2 | 3 |

**Anexo M.** EU Clinical trials register (https: //www.clinicaltrialsregister.eu)

| **Terms** | **#** |
| --- | --- |
| Infliximab OR "MAb cA2" OR "Monoclonal Antibod*" OR "Monoclonal Antibody cA2" OR Remicade OR "Anti-TNF-α" | 1 |
| switch OR interchange OR substitute | 2 |
| #1 AND #2 | 3 |

**Annex N.** Clinical trials.gov (http://www.clinicaltrials.gov)

| **Términos** | **#** |
| --- | --- |
| Infliximab OR "MAb cA2" OR "Monoclonal Antibod*" OR "Monoclonal Antibody cA2" OR Remicade OR "Anti-TNF-α" | 1 |
| switch OR interchange OR substitute | 2 |
| #1 AND #2 | 3 |
